# Mesozoic fossils (>145 Mya) suggest the antiquity of the subgenera of *Daphnia *and their coevolution with chaoborid predators

**DOI:** 10.1186/1471-2148-11-129

**Published:** 2011-05-19

**Authors:** Alexey A Kotov, Derek J Taylor

**Affiliations:** 1 AN Severtsov Institute of Ecology and Evolution, Leninsky Prospect 33, Moscow 119071, Russia; 2 Department of Biological Sciences, The State University of New York at Buffalo, Buffalo, NY 14260, USA

## Abstract

**Background:**

The timescale of the origins of *Daphnia *O. F. Mueller (Crustacea: Cladocera) remains controversial. The origin of the two main subgenera has been associated with the breakup of the supercontinent Pangaea. This vicariance hypothesis is supported by reciprocal monophyly, present day associations with the former Gondwanaland and Laurasia regions, and mitochondrial DNA divergence estimates. However, previous multilocus nuclear DNA sequence divergence estimates at < 10 Million years are inconsistent with the breakup of Pangaea. We examined new and existing cladoceran fossils from a Mesozoic Mongolian site, in hopes of gaining insights into the timescale of the evolution of *Daphnia*.

**Results:**

We describe new fossils of ephippia from the Khotont site in Mongolia associated with the Jurassic-Cretaceous boundary (about 145 MYA) that are morphologically similar to several modern genera of the family Daphniidae, including the two major subgenera of *Daphnia*, i.e., *Daphnia *s. str. and *Ctenodaphnia*. The daphniid fossils co-occurred with fossils of the predaceous phantom midge (Chaoboridae).

**Conclusions:**

Our findings indicate that the main subgenera of *Daphnia *are likely much older than previously known from fossils (at least 100 MY older) or from nuclear DNA estimates of divergence. The results showing co-occurrence of the main subgenera far from the presumed Laurasia/Gondwanaland dispersal barrier shortly after formation suggests that vicariance from the breakup of Pangaea is an unlikely explanation for the origin of the main subgenera. The fossil impressions also reveal that the coevolution of a dipteran predator (Chaoboridae) with the subgenus *Daphnia *is much older than previously known -- since the Mesozoic.

## Background

The timescale of the evolution of some of the most successful freshwater microcrustacean groups such as copepods and cladocerans is poorly known or controversial. Both groups are comprised of small and often fragile species, whose fossilized body parts might easily be overlooked. Copepods appear be predisposed to weak fossilization as they are extremely rare in the subfossil and fossil record and apparently only preserved under very unusual circumstances such as oil seeps [1]. Tasch [2], however, reasoned (after conducting drying experiments of cladocerans on pond mud) that body parts of cladocerans should be well-preserved in freshwater sediments. Of course, the subfossils of cladoceran ephippia (modified moulting exuviae containing resting eggs) and heavily chitinized body parts are common in lacustrine sediments -- fossilized cladoceran resting eggs had already been reported by 1968 from the Pliocene, the Miocene and the Eocene [3-6]. Tasch [2] "hunted thoroughly" for cladoceran fossils without success in Paleozoic freshwater sediments and later predicted that cladocerans probably arose during the Mesozoic. Proposals of Paleozoic records of cladocerans [7] turned out to be of non-cladoceran origin [8], supporting Tasch's hypothesis of a later origin for cladocerans. Later, authentic Mesozoic cladoceran fossils were discovered from several sites [8-14].

Still, for the cladoceran genus *Daphnia *O. F. Muller, 1785 (Cladocera: Anomopoda), there is a lack of reliable calibration points from either geographic or from fossil evidence. The present day geographic domination of former Gondwanaland regions by the subgenus *Daphnia *(*Ctenodaphnia) *Dybowski et Grochowski, 1895 and of former Laurasian regions by the subgenus *Daphnia *(*Daphnia*), and the reciprocal monophyly of each subgenus [15-17] could indicate an ancient vicariance event in the early Cretaceous [18,19]. But, the north-south hemispheric dichotomy of the subgenera in extant *Daphnia *is unapparent in the sparse fossil record -- every Cenozoic record with fossilized ephippia from the northern hemisphere reports *Ctenodaphnia*-like fossils (China, Germany, Spain, and USA) [4,20,21]. Moreover, *Daphnia ephemeralis *(Schwartz et Hebert, 1985), the oldest member of the presumed Gondwanaland clade [15,17] is restricted to former Laurasia. The oldest fossils of the subgenus *Daphnia *are known from the northern hemisphere only at the German Lake Messel site (Eocene) where fossils of *Ctenodaphnia *are also recorded. Smirnov [11] assigned impression fossils of Mesozoic ephippia to the genus *Daphnia *(or "*Daphnia*-type"), but the subgenus was unassigned.

A deeper understanding of the timescale of daphniid evolution has implications beyond biogeography and systematics. Daphniids have become the model taxon for ecological genomics and the evolution of inducible defenses [22-24]. The best studied inducible defenses involve responses of daphniids to chemical cues resulting from predation by the phantom midge larvae (Chaoboridae) [25]. Numerous phenotypic responses (morphology, life-history, and behavior) have been reported from daphniids. Initial studies indicate that genomic control of inducible defenses is polygenic and complex [23,26,27], with a majority of responsive genes having no known homologs [23]. Indeed species of both major subgenera of *Daphnia *appear to possess complex genomic adaptations to predation by chaoborids [24,25]. The phantom midge family (Chaoboridae) has an excellent Mesozoic fossil record in Asian lake fossil beds with thousands of specimens known from at least five locations [28]. Does the timescale of co-evolution between *Daphnia *and phantom midges extend to the Mesozoic?

Without reliable independent calibrations, molecular estimates of the divergence of *Daphnia *have also been predictably conflicting. In general, estimates from mitochondrial genes appear consistent with a Mesozoic origin of the subgenera [17,29-31]. Nuclear estimates of divergence, however, have been dramatically lower than estimates from mitochondrial genes, a phenomenon that has been attributed to differences in the effective population sizes and responses to selection for the respective genomes. Nuclear DNA estimates of divergence based on observed mutation rates of *Drosophila *and *Caenorhabditis elegans *have yielded divergence times at 7.6 MY for the subgenera of *Daphnia *[32]. This young estimate of divergence requires that the mitochondrial DNA estimates and the conclusions of Richter and Wedmann [6], who assigned damaged ephippia from fish coprolites in Eocene deposits (about 47 Myr) to *Daphnia *(*Daphnia*) *pulex *and *Daphnia *(*Ctenodaphnia*) *magna*, to be spurious [33].

**Figure 1:**
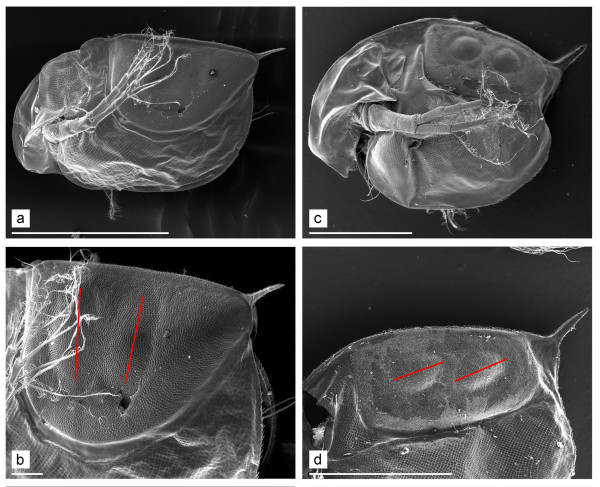
**SEMs of ephippial females of extant *Daphnia***. a-b. *Daphnia *(*Daphnia*) *pulex*, general view of ephippial female and ephippium. c-d. *Daphnia *(*Ctenodaphnia*) *magna*, general view of ephippial female, ephippium and its sculpture. Red lines show the orientation of the egg axes. White scale bars: 1 mm for a, c-d; 0.1 mm for b.

**Figure 2:**
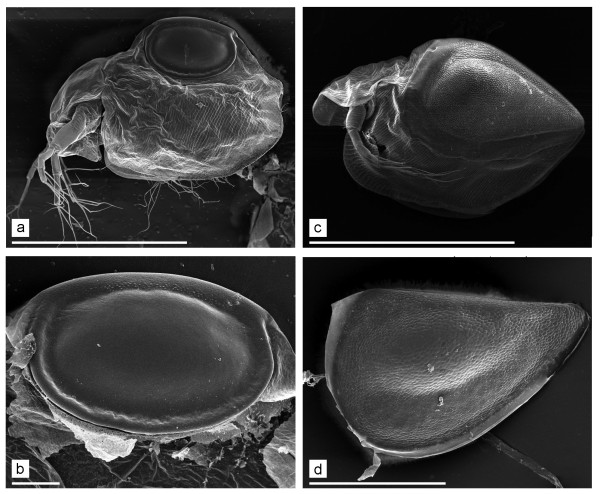
**Single-egged ephippial females of *Daphnia *and *Simocephalus***. a-b. *Daphnia (Ctenodaphnia) pusilla*, general view of ephippial female and ephippium. c. *Simocephalus exspinosus*, ephippial female. d. *Simocephalus vetulus*, ephippium. Scales: 1 mm for a, c; 0.1 mm for b, d.

More evidence is needed to resolve the existing profound disagreements of the timescale of evolution for the genus *Daphnia*. Importantly, the ephippium has been identified as possessing important diagnostic characters for the subgenera [19]. In *Daphnia *(*Daphnia*) the ephippium is sub-triangular in shape (because the anterior half of the ephippium is deeper than the posterior half), with axes of two eggs perpendicular or sub-perpendicular to the dorsal margin (Figure 1a-b). In contrast, the ephippium of *D*. (*Ctenodaphnia*) Dybowski & Grochowski, 1895 is usually D-shaped, with the axes of eggs being sub-parallel to dorsal margin (Figure 1c-e). The third subgenus, *D. (Australodaphnia) *Colbourne, Wilson et Hebert, 2006 represented now only by a single species, *Daphnia occidentalis *Benzie, 1986, has only a single egg in the ephippium [34]. We note that some extant *Ctenodaphnia *(Figure 2a-b) and all other daphniid genera, i.e. *Simocephalus *(Figure 2c-d) also have a single large egg in the ephippium.

Inspired by the relatively undamaged impression fossils of ephippia reported by Smirnov [11] from Mesozoic Mongolian sediments, we examined existing and new samples from this site. We report that there is fossil evidence for an ancient Mesozoic divergence of the subgenera of *Daphnia*. The evidence extends the age of the subgenera by about 100 MY from existing fossils and by about 138 MY from nDNA mutation rate estimates. Further, the co-occurrence of fossils from the subgenera and from chaoborids indicates a coevolutionary history of predator and prey taxa that also dates to the Mesozoic.

## Results

Limestone fragments with clear impressions were assigned specimen numbers and stored at the Palaeontological Institute of Russian Academy of Sciences. The number of impression fossils per fragment is given in parentheses below. Fragments with numbers 4307/2001 - 4307/2040 were previously discovered by Smirnov [9] and re-examined here; fragments with subsequent numbers were newly discovered by AAK and described below.

### Daphnia (Daphnia) sp.?

**Material examined**. 4307/2046 (2).

**Figure 3:**
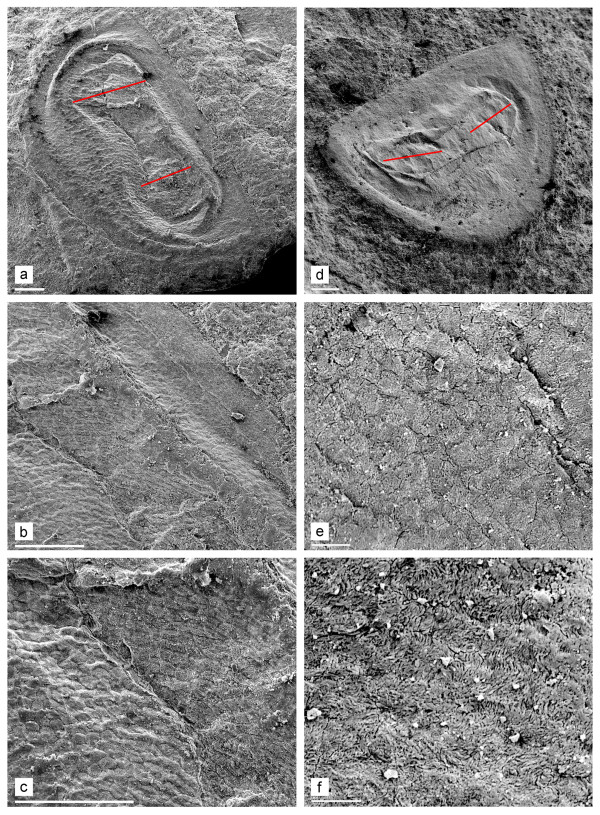
**SEMs of Mesozoic ephippia of *Daphnia *from Khotont, Mongolia**. a-c. Putative ephippium of *Daphnia *(*Daphnia*) from fragment 2046, its dorsal portion and reticulation. Note that the anterior half of the ephippium is deeper than the posterior half, giving a sub-triangular shape. d-f. Ephippium of *Daphnia *(*Ctenodaphnia*) from fragment 2018, reticulation and fine sculpture of valve. Red lines show the putative orientation of the egg axes. White scale bars: 0.1 mm for a, d; 0.01 mm for b-c, e-f.

**Description**. Lengths are 0.86 mm and 1.03 mm. The ephippium is sub-triangular, relatively high (height/length = 0.59 and 0.63), with an almost straight dorsal margin. The caudal needle or spine is missing and there is a widely rounded antero-dorsal and postero-dorsal angle (Figure 3a). The dorsal portion of the ephippium is a non-reticulated, heavily-chitinized plate (Figure 3b). Much of the ephippial surface has inflated reticulation (Figure 3c). The axes of the two eggs are almost perpendicular to the dorsal margin.

### Daphnia (Ctenodaphnia) sp

**Material examined**. 4307/2004 (2); 4307/2007 (1); 4307/2018 (1); 4307/2024 (1); 4307/2025 (1); 4307/2026 (1); 4307/2033 (1); 4307/2042 (4); 4307/2044 (2); 4307/2048 (1); 4307/2049 (2).

**Figure 4:**
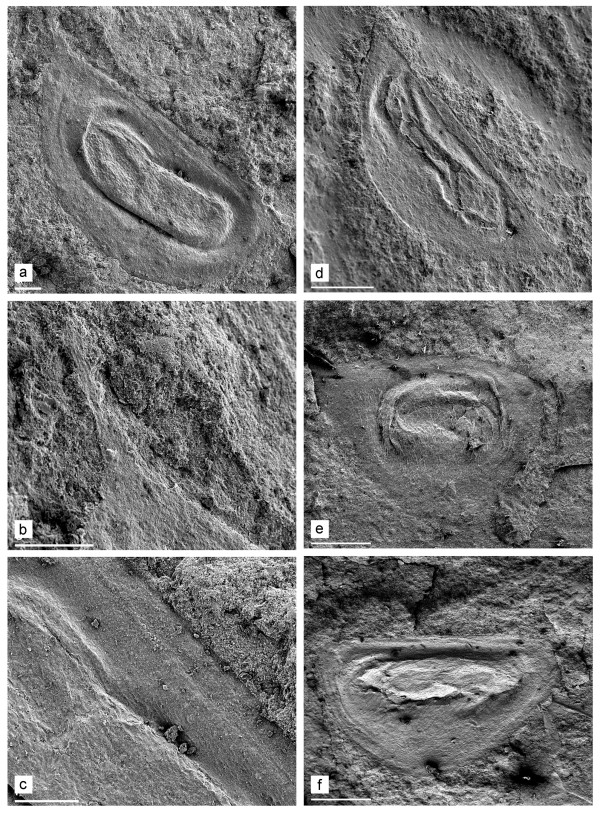
**SEMs of Mesozoic daphniid ephippia from Khotont, Mongolia**. a-c. *Daphnia *(*Ctenodaphnia*) from fragment 2048, its caudal needle and dorsal portion. d. *Daphnia *(*Ctenodaphnia*) from fragment 2044. e. Unknown daphniid from fragment 2009. f. *Simocephalus *from fragment 2026. Scales: 0.1 mm for a, d-f; 0.01 mm for b-c.

**Description**. Length 0.97-1.17 mm. The ephippia have a varying relative height (height/length = 0.50-0.69), with a dorsal plate, and axes of eggs parallel to the dorsal margin. We found size differences among the ephippia of *Daphnia *(*Ctenodaphnia*), but the material is too scarce to designate species. Larger ephippia usually have a widely rounded antero-dorsal angle and a distinct caudal needle at the postero-dorsal angle. The surface has a well-developed sculpture, with patterns varying among individuals (Figures 3d-f, 4a-c). In contrast, the smaller ephippia have acute antero-dorsal and postero-dorsal angles lacking a caudal needle and surface sculpturing (Figure 4d).

### Daphniidae gen. sp. 1

**Material examined**. 4307/2009 (1).

**Redescription**. Length 1.02 mm. The ephippium is high, with a slightly convex dorsal margin, a widely rounded anterior margin and a triangular posterior margin (Figure 4e). The entire surface is covered with dorso-ventral striation. The axis of a single egg is located parallel to the dorsal margin, and the egg chamber occupies about half of the ephippium length.

**Comments**. This ephippium was named "of *Moina*-type" by Smirnov [11], but the generic determination is uncertain. Most probably, this is a daphniid ephippium, but a very primitive one. Recent species of *Moina *have ephippia with well-developed sculpture of different types [5]. In contrast, the ephippium from fragment 4307/2009 is only striated. Other daphniids, namely *Ceriodaphnia *and some species of *Daphnia *(*Ctenodaphnia*) (see Figure 2a-b) lay monoegged ephippia (in the case of *Daphnia *they are sometimes lacking a caudal needle or spine). So, the generic position of this ephippium is unclear.

### Simocephalus sp

**Material examined**. 4307/2026 (1); 4307/2042 (1).

**Redescription**. The ephippium is elongated, with a straight dorsal margin, a widely rounded anterior margin and a fluently narrowing posterior portion. The surface is finely reticulated (Figure 4f). The axis of a single, elongated egg is located parallel to the dorsal margin. The egg chamber occupies more than 2/3 of ephippium length. Length: 1.02 and 1.10 mm.

**Comments**. The ephippium from fragment 4307/2026 was justifiably named "of *Simocephalus*-type" by Smirnov [11], indeed, it is quite similar to the ephippia of recent species from this genus (Figure 2c-d). Among daphniids, the ephippium of *Simocephalus *is quite unique in possessing a fluently narrowing posterior portion and a large, elongated resting egg [35]. Although ephippia of recent species normally have well-developed sculpturing, weak sculpturing of the Mesozoic ephippia could be an artefact of impression fossil formation or of among-species variation.

## Discussion

Our finding of ephippia from the Khotont site that are morphologically similar to the common subgenera of *Daphnia, D. (Daphnia) *and *D*. (*Ctenodaphnia*), indicates that these two subgenera potentially existed at the J-K boundary (at least 145 Mya). Our results also confirm the finding of Smirnov [11] that additional daphniid genera (i.e. *Simocephalus*) are present at this boundary. The fossils of Khotont likely extend the fossil record of the subgeneric divergence of *Daphnia *by about 100 MY.

**Figure 5:**
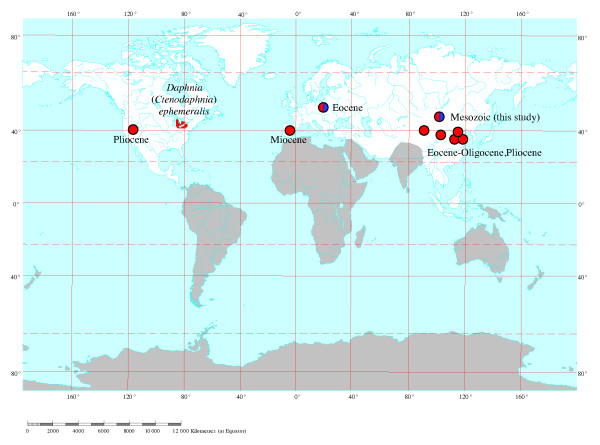
**Global map showing fossil records of the genus *Daphnia *and the antiquity of the subgenus *Ctenodaphnia *in the former Laurasia**. Circles indicate fossil records of *Daphnia *colored by subgenus (*Ctenodaphnia *is red and *Daphnia *s. str. is blue). The grey shaded continents indicate the former Gondwanaland regions and the unshaded regions represent the former Laurasia regions. Red shading in North America indicates the present day distribution of the basal *Ctenodaphnia *from phylogenetic and morphological information.

The detection of the subgenus *Ctenodaphnia *in the northern hemisphere during the J-K boundary poses a problem for the hypothesis of vicariance by the break up of the supercontinent Pangaea. The continental breakup had occurred by 155 MYA forming Laurasia and Gondwana, but the two lineages apparently co-occurred far from the presumed dispersal barrier in northern Laurasia by about 145 MYA. Under the "out of Gondwanaland" requirement for the current vicariance hypothesis, the subgenus *Ctenodaphnia *would have had to breach a more severe oceanic dispersal barrier than contributed to initial speciation, and colonize the most distant continent from Gondwanaland. This requires the unlikely transcontinental dispersal before the major dispersal vector of daphniids (modern birds) had evolved. Instead, the current fossil and phylogenetic evidence indicates that the break up of Pangaea is not associated with the origins of the main subgenera of *Daphnia*. *Ctenodaphnia *and *Daphnia *appear to have had a presence in Laurasia since the early Mesozoic (Figure 5). *Ctenodaphnia *are known from throughout much of the present northern hemisphere and one of the northern endemics, *Daphnia ephemeralis*, is likely the oldest known member of the *Ctenodaphnia*, with a basal position in the *Ctenodaphnia *phylogeny. *D. ephemeralis *also has a primitive morphology for the subgenus with the female having: (1) a very thick, almost rounded body; (2) no caudal needle in adults; (3) a rounded fornix; (4) a weakly developed rostrum; and (5) no posterior mid-line depression on the head shield and, as a result, a W-shape of the dorsocephalic suture (see [19] for terminology). *Ctenodaphnia *fossils have been found from the Pliocene of Nevada, USA, the Miocene of Spain, the Pliocene, Oligocene and Eocene of China, the Eocene of Germany, and here from the Mesozoic of Mongolia (Figure 5). No fossil *Ctenodaphnia *species are yet known from former Gondwanaland. Although the geographic distribution of known fossils and "basal" species appears inconsistent with Gondwanaland origins for *Ctenodaphnia*, the role of extinction and differential fossilization in the formation of these patterns is unknown. It is clear that the age of our new fossils together with existing fossil evidence makes the hypothesis of Pangaea-related vicariance less likely.

Our results provide a case where both molecular dating (save the mtDNA estimates) and current biogeography appear to have been misleading regarding the timescale of evolution. Haag et al. [32] discuss potential reasons (error associated with estimates of mutation rate, number of generations per year, and population subdivision) for discrepancies between their nDNA dating and the existing mtDNA clock estimates (and now fossil estimates). The dominance of the southern hemisphere by the *Ctenodaphnia *when most *Daphnia *appear capable of among-continent dispersal remains a mystery. Presently, species of *Daphnia *s. str. prefer large lakes, while members of *D*. (*Ctenodaphnia*) occur most often in small temporary water bodies (although there are exceptions from this general rule). If this preference evolved early in the differentiation of the subgenera, the greater preference for non-lacustrine and temporary habitats in the *Ctenodaphnia *compared to *Daphnia *combined with priority effects are plausible explanations for the differential success of *Ctenodaphnia *in the southern hemisphere (see also [17]). In the large Mesozoic lake Khotont both subgenera were found together. But this finding does not mean that they cooccurred in a stable fashion. We cannot rule out the possibility that ephippia were introduced from the surrounding (permanent or temporary) lentic water bodies during some high water periods, or by some lotic waters. Most commonly, the contents of such small water bodies are not fossilised -- we have palaeontological information from cladocerans largely from lakes.

We assumed that ephippial characters that represent subgenera of extant species also reflect the same subgenera in Mesozoic taxa. However, it is plausible that ephippial characters from extant subgenera are non-diagnostic in Mesozoic taxa. A scenario of independent origins for ephippial characters or ancestral polymorphisms might result in Mesozoic ephippia being uninformative for subgeneric reconstruction. However, the multiple origins of characters requires more evolutionary steps than character stability, and the ancestral polymorphisms scenario requires presumably independent diagnostic characters (shape and egg axis characters) to sort together. Ideally, more characters will be found from Mesozoic fossils of *Daphnia *that might address the hypothesis of evolutionary stability for diagnostic ephippial characters. Numerous cladocerans lacking an ephippium are reported for the same locality, namely, extinct prochydorids of three different species [11,14] and a single antenna II of an undetermined ctenopod [12]. However, no complete adult or body part beyond ephippia is known from the daphniids of Khotont. The fact of poor preservation of daphniids compared to other cladocerans in bottom deposits is well-known [10]. Recently Richter and Baszio [36] demonstrated that the fine structure of Eocene daphniids, including limb structure, could be studied from fish coprolites -- a type of fossil that is presently unknown from the Khotont. It seems clear that impression fossils yield well-preserved ephippia, but intact daphniid specimens will probably be best sought in fish coprolites from Mesozoic localities.

Remarkably, daphniids in Lake Khotont were found together with numerous (587 impressions were found) fossils of the dipteran (Chaoboridae) predator of daphniids [37,38]. Our results provide evidence that the phenotypic array of inducible defenses in *Daphnia *is more than a rapidly evolved response to predation. This important predator-prey group has been coevolving in freshwater for at least 145 MY. We expect that the antiquity of daphniidchaoborid coevolution will be reflected in both predator and host genomes.

## Conclusions

Our findings indicate that the main subgenera of *Daphnia *could be much older than previously known from fossils (at least 100 MY older) or from nuclear DNA estimates of divergence. However, the results showing co-occurrence of the main subgenera far from the presumed Laurasia/Gondwanaland dispersal barrier shortly after formation suggests that vicariance from the breakup of Pangaea is an unlikely explanation for the origin of the main subgenera. The fossil impressions also reveal that the coevolution of a dipteran (Chaoboridae) predator with the subgenus *Daphnia *has been occurring much longer than previously known -- since the Mesozoic.

## Methods

Numerous limestone fragments were collected in the northern slope of Ukha Mount, 6 km W of Somon Khotont, Ara-Khangay Aymag (=Region) of Mongolia by the Expedition of the Palaeontological Institute of Russian Academy of Sciences (PIN) in 1980. Fragments from Khotont were taxonomically identified by the staff of PIN. Relative dating based on the examination of several thousand index fossils of insects (dipteran insects, for example, were represented by 948 impressions), assigned the present fragments to the Jurassic/Cretaceous boundary [38] at about 145 Mya. Some cladocerans from this locality have been previously studied [11,12,14].

For the present work, fourteen limestone fragments with 24 comparatively clear impressions of daphniid ephippia, seen by Smirnov [11] or subsequently found on fragments with other animal impressions, were selected. Micrographs were taken using the scanning electron microscope CAM SCAN MB2300 after coating with gold.

## Authors' contributions

AAK and DJT conceived the study and co-wrote the paper. Both authors read and approved the final manuscript.
